# Super-resolution reconstruction of late gadolinium enhanced MRI from multiple views

**DOI:** 10.1186/1532-429X-16-S1-O40

**Published:** 2014-01-16

**Authors:** Qian Tao, Oleh Dzyubachyk, Dirk Poot, Hildo J Lamb, Katja Zeppenfeld, Boudewijn PF Lelieveldt, Rob J van der Geest

**Affiliations:** 1 Department of Radiology, Leiden University Medical Center, Leiden, Netherlands; 2 Department of Cardiology, Leiden University Medical Center, Leiden, Netherlands; 3 Departments of Radiology and Medical Informatics, Erasmus Medical Center, Rotterdam, Netherlands; 4 Intelligent Systems Department, Delft University of Technology, Delft, Netherlands

## Background

Image resolution is a crucial factor for myocardial scar quantification from late gadolinium enhanced (LGE) MR. The conventional usage of anisotropic short-axis LGE volumes may result in overestimation of the scar size, in particular at the gray zone, due to partial volume effect (PVE). This may impact the diagnostic accuracy of LGE in clinical applications.

## Methods

A group of 37 post-infarction patients with ventricular tachycardia (VT), who underwent both MRI and a VT catheter ablation procedure with electro-anatomical voltage mapping (EAVM), were included. LGE imaging was performed using a 3D breath-hold inversion recovery turbo-field echo sequence with full ventricular coverage in three views: short-axis (SA), two-chamber, and four-chamber. The three LGE sequences were combined as follows: 1) The inter-scan heart motion was compensated by a joint localized gradient-correlation-based volume registration scheme; 2) The registered volumes were combined into an isotropic volume by the super-resolution reconstruction (SRR) technique. The SRR reconstructed volumes were compared to conventional SA volume using the following measures: the full-width-half-maxima (FWHM)-identified myocardial scar size in terms of core zone and gray zone; the agreement between the scar zones and the gold-standard EAVM data. The agreement was evaluated by the voltage distribution of the mapping points which were back projected onto the normal myocardium, scar core zone and gray zone.

## Results

**Figure 1:**
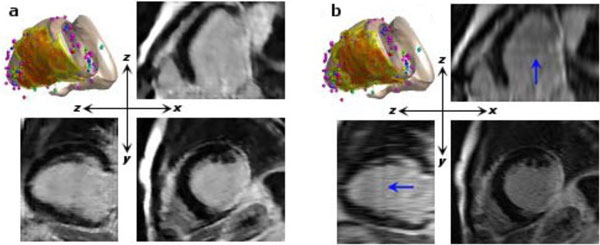
**a. The 3D reconstruction and orthogonal planes from the SRR volume**. b. The 3D reconstruction and orthogonal planes from the SA volume. Higher quality in SRR is achieved at z-x and z-y planes.

**Figure 2:**
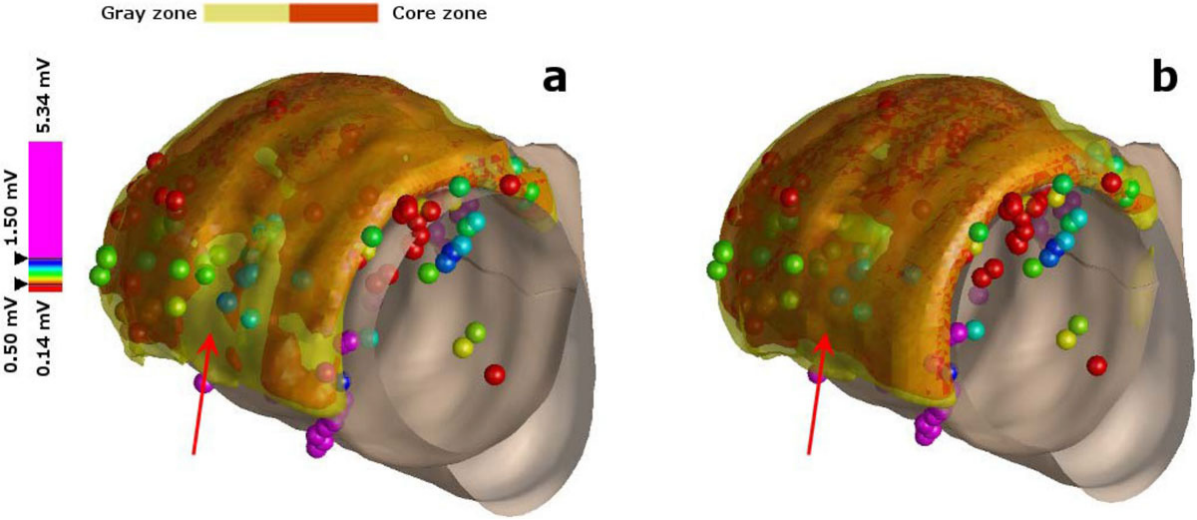
**The 3D reconstruction of the myocardial scar, including the core (orange) and gray (yellow) zones, superimposed by the EAVM data**. The EAVM colors indicate: healthy myocardium (pink), core scar (red), and gray zone (intermediate colors). a is from the SRR volume, and b is from the SA volume.

The SRR volume achieved increased resolution in the through-plane direction (Figure 1). Compared to the SA volume, the SRR volume resulted in significantly reduced scar size estimation for both scar core zone (9.6 ± 7.8 ml vs. 8.7 ± 8.3 ml, p < 0.05) and gray zone (11.9 ± 8.2 ml vs. 7.9 ± 6.0 ml, p < 0.05). Average EAVM bipolar voltage for SRR and original SA volume were in normal myocardium 3.7 ± 3.3 mV versus 3.8 ± 3.3 mV (p = NS); in scar core 0.8 ± 0.6 mV versus 1.0 ± 1.0 mV (p = 0.04) and scar gray zone 1.2 ± 0.8 mV versus 1.6 ± 1.7 mV (p = 0.005). SRR volumes showed better agreement with EAVM (Figure 2).

## Conclusions

We proposed a method to reconstruct a high-resolution isotropic LGE MR volume from three routinely acquired anisotropic views. Improving the through-plane resolution of LGE leads to more accurate myocardial scar assessment with reduced PVE, especially at the clinically significant gray zone.

## Funding

The work was financially supported by the Dutch Technology Foundation (STW) and EU ITEA2-09039.

